# Integrated Transcriptome Analysis Reveals the Impact of Photodynamic Therapy on Cerebrovascular Endothelial Cells

**DOI:** 10.3389/fonc.2021.731414

**Published:** 2021-11-22

**Authors:** Yanyan He, Lin Duan, Haigang Wu, Song Chen, Taoyuan Lu, Tianxiao Li, Yingkun He

**Affiliations:** ^1^ Department of Cerebrovascular Disease, Henan Provincial People’s Hospital, Zhengzhou University People’s Hospital, Henan International Joint Laboratory of Cerebrovascular Disease, Zhengzhou, China; ^2^ School of Life Sciences, Henan University, Kaifeng, China; ^3^ Translational Research Institute, Henan Provincial People’s Hospital, Zhengzhou University, Academy of Medical Science, Zhengzhou, China

**Keywords:** photodynamic therapy, RNA seq, blood-brain barrier, gliomas, endothelial dysfunction

## Abstract

Blood vessels in the brain tissue form a compact vessel structure and play an essential role in maintaining the homeostasis of the neurovascular system. The low dosage of photodynamic intervention (PDT) significantly affects the expression of cellular biomarkers. To understand the impact of photodynamic interventions on cerebrovascular endothelial cells, we evaluated the dosage-dependent impact of porfimer sodium-mediated PDT on B.END3 cells using flow cytometer, comet assay, RNA sequencing, and bioinformatics analysis. To examine whether PDT can induce disorder of intracellular organelles, we did not observe any significance damage of DNA and cellular skeleton. Moreover, expression levels of cellular transporters-related genes were significantly altered, implying the drawbacks of PDT on cerebrovascular functions. To address the potential molecular mechanisms of these phenotypes, RNA sequencing and bioinformatics analysis were employed to identify critical genes and pathways among these processes. The gene ontology (GO) analysis and protein-protein interaction (PPI) identified 15 hub genes, highly associated with cellular mitosis process (*CDK1*, *CDC20*, *MCM5*, *MCM7*, *MCM4*, *CCNA2*, *AURKB*, *KIF2C*, *ESPL1*, *BUB1B*) and DNA replication (*POLE2*, *PLOE*, *CDC45*, *CDC6*). Gene set enrichment analysis (GSEA) reveals that *TNF-α/NF-κB* and *KRAS* pathways may play a critical role in regulating expression levels of transporter-related genes. To further perform qRT-PCR assays, we find that *TNF-α/NF-κB* and *KRAS* pathways were substantially up-regulated, consistent with GSEA analysis. The current findings suggested that a low dosage of PDT intervention may be detrimental to the homeostasis of blood-brain barrier (BBB) by inducing the inflammatory response and affecting the expression of surface biomarkers.

## Introduction

Blood circulation system plays a critical role in maintaining the homeostasis physiology and supplying the essential nutrients to the targeted organisms or tissue (1). In the nervous system, the blood-brain barrier (BBB) was composed of endothelial cells and other types of cells, for example pericytes and neuron end-foot, to form the compact vascular system (2, 3). The compact BBB can blockade most of the unnecessary molecules to be diffused to the nervous system, and positively pump harmful metabolism waste into blood circulation (4). However, dysfunction or impairment of BBB involves or promotes some pathological processes, such as promoting neurodegenerative inflammation in the brain (5, 6), breakdown of BBB in stroke (7), and psychosis due to BBB disorder (8). Moreover, cerebrovascular BBB could be stimulated by various exogenous factors, such as vascular endothelial growth factor (VEGF) (9, 10), to promote angiogenesis and support the growth of brain tumor (11) during tumorigenesis. These results indicated that any unexpected interventions might lead to a severe stimulus that would disrupt the homeostasis of the neuron-blood system. Thus, exploring the stimulus response of vasculature after any therapeutic interventions, including the damage and biological impact on the biological functions, is imperative.

Photodynamic therapy (PDT) has been widely utilized in the glioblastoma multiforme (GBM) therapy, including the interstitial PDT (iPDT) and post-PDT (12). PDT requires three essential components: oxygen, radiation light, and photosensitizers (13). The intrinsic mechanism of PDT is the interaction between cellular components and reactive oxygen species (ROS), causing damage of cellular components and leading to cell apoptosis (14). After receiving PDT intervention, the overall survival (OS) of GBM patients can be prolonged from 15 months to 27 months (15). The potential reasons for improving the OS of GBM patients may be attributed to increased permeability of chemotherapeutic drugs (16) across the BBB. These results from clinical reports implied that the PDT intervention might strongly stimulate the BBB endothelial cells for specific responses. To further identify and explore the pivotal changes in endothelial cells after PDT intervention may provide more necessary information to guide the clinical application of PDT on GBM therapy.

The interventional effects of PDT on the endothelial cells are decided by types of photosensitizers and specific cell lines (17, 18). Hitherto, many types of photosensitizers have been clinically approved for disease therapy; for example, 5-aminolevulinic acid (5-ALA) (19), hematoporphyrin derivative (HPD) (20), and porfimer sodium (21, 22). Hemoporfin-mediated photodynamic therapy induces cellular autophagy to prevent cellular apoptosis (23). Aloe-emodin-mediated PDT activates the MAPK signaling pathway on HUVECs to inhibit angiogenesis and cell metastasis (24); verteporfin-mediated PDT promotes the expression of vascular endothelial growth factor (VEGF), VEGF receptor (VEGFR)-3, and pigment epithelium-derived factor (PEDF) (25); a low dose of photofrin-mediated PDT increased the expression of VEGF and promoted endothelial cell proliferation in normal brain (26). However, the effect of PDT on the cerebrovascular endothelial cells, especially at transcriptomic levels, might improve a emerging scope for evaluating the impact of PDT on the nervous system.

Herein, we explored the biological impact of the photodynamic intervention on endothelial cells, including apoptosis, DNA damage, cellular skeleton, and expression levels of critical transporter-related genes. Then, we utilized RNA-seq to analyze the biological impact of porfimer sodium-driven photodynamic intervention on the cerebrovascular endothelial cells of the mouse (B.END3 cells). We identified 187 and 2976 differentially expressed gene (DEG) depending on two different interventional dosages, respectively. Bioinformatics analysis using gene ontology (GO), gene set enrichment analysis (GSEA), and KEGG gene sets identified critical pathways that were confirmed by quantitative real-time PCR. Thus, the current study provides additional information about the transcriptome changes in the cerebrovascular endothelial cells during the PDT process and the scope to further evaluate the impact of the photodynamic intervention on BBB homeostasis.

## Methods

### Cell Culture

Rat endothelial B.END3 cells were obtained from the Cell Bank of the Chinese Academy of Sciences and cultured in DMEM medium (SH30022.01B, Gibco, USA) with 10% fetal bovine serum (FBS; SH30084.03, HyClone, USA) and 100 U/mL penicillin-streptomycin (Gibco, Cat. 15140122, USA) under 5% CO_2_ at 37°C. When the B.END3 cells formed the monolayer, the photosensitizer Porfimer sodium (300 μg/mL) was added to the medium and incubated for 90 min. Then, the B.END3 cells received the laser exposure (635 nm with 100 mW/cm^2^) and the light doses at 10 J/cm^2^ and 20 J/cm^2^, respectively.

### RNA Sequencing

After PDT intervention, the cells were lysed, collected, and stored at -80°C until delivered to the Tianjin Novogene Bioinformatic Technology Co., Ltd for further analysis. The whole-genome transcriptome profiling was examined by RNA sequencing process: sequencing on the Illumina Hiseq2500 using 150 bp paired-end reads (6.0 G of throughput). The RNA sequencing data have been deposited to NCBI GEO database (GEO accession cat. GSE172198).

Total RNA was extracted from the cell lysis samples using Total RNA Extraction Kit (R1200/100T, Solarbio Life Sciences, China) and then reverse-transcribed into cDNA using the PrimeScript™ RT MasterMix (Perfect Real Time) (TaKaRa, Japan). The cDNA was used for qPCR using TB Green^®^ Premix Ex Taq™ (Tli RNaseH Plus) (TaKaRa) with gene-specific primers, and the data were normalized against *β-actin* as the control. PCR primers are listed in **Table S1**.

### Bioinformatics Analysis

Before further analysis, the RNA data were aligned against the mouse genome (GRCm39, GenBank assembly accession: GCA_000001635.9) and deposited in NCBI GEO database. The GO function analysis was performed using g:profilter website (https://biit.cs.ut.ee/gprofiler/gost), in which the “ordered query” was selected and other parameters were set as default. The protein-protein interaction (PPI) network was assessed using the STRING website (https://string-db.org/), while the minimum required interaction score was set as the highest confidence (0.900) and kmeans clustering was set as 5. The PPI network was re-generated using Cytoscape (version 3.6.0) with a circular layout. GSEA was performed using GSEA software (v4.1.0) with the molecular signature database obtained from the GSEA website (http://www.gsea-msigdb.org/gsea/msigdb/index.jsp); number of permutations was set as 10000; no collapse was aligned to gene symbols; permutation type was set as gene set, and other parameters were set as default. The function-related information of above-mentioned genes in this study was obtained from the GENE section of NCBI (https://www.ncbi.nlm.nih.gov/). Transcription factors of DEGs were obtained from TRRUST database (https://www.grnpedia.org/trrust/).

All the statistical results and figures were generated using GraphPad_Prism 5.0, and the Venn diagram was obtained from the Van der Peer Lab bioinformatics website (http://bioinformatics.psb.ugent.be/webtools/Venn/). For GSEA analysis, the significance of enriched pathways was set as |Normal Enrichment Score|>1.0 and NORM p-value < 0.05 and FDR q-value <0.05. For other analyses, p-value < 0.05 was considered statistically significant.

### Comet Assay

After PDT intervention, 1×10^5^ cells were digested, purified, and mixed with 30 μL of low-melting-point agarose (LMPA, 1% DMEM solution). Then, this cell solution was dropped on the glass slide to form a thin film and cooled for 10 min using ice to allow solidification. Then, an additional 75 μL of LMPA (1% DMEM solution) was dropped on this glass slice as the top layer, and the process was repeated. These samples were dipped in the lysis solution (containing 10 mM Tris-HCl, 2.5 M NaCl, 100 mM Na_2_EDTA, 1% Triton X-100) overnight. The DNA sample was unwound for 20 min in the alkaline electrophoresis solution and electrophoresis performed for 20 min (voltage 1 V/cm and current 300 mA). Finally, these samples were stained using ethidium bromide (EB; 100 μL, 20 μg/mL). The images of DNA damage were obtained under Zeiss 880 confocal microscopy.

### Immunofluorescence

For p53 translocation assay and actin staining assay, B.END3 cells were seeded on glass coverslips and then fixed in 4% paraformaldehyde at room temperature for 10 min. The preparation protocol was performed using standard processes described previously (27, 28). p53 (MA5-12557), Alexa Fluor™ 647 (Invitrogen™, A20186), and Alexa Fluor™ 488 Phalloidin (Invitrogen™, A12379) were purchased from ThermoFisher Scientific.

### Statistical Analysis

Statistical examination and image preparation of assays were performed using GraphPad Prism 5.0 software (GraphPad Software Inc.). Student’s *t*-test was performed: *, *p* < 0.05; **, *p* < 0.01; ***, *p* < 0.001; ****, *p* < 0.0001; ***ns***, no significance.

## Results and Discussion

### Biological Impact of the Photodynamic Intervention on Endothelial Cells

**Figure 1:**
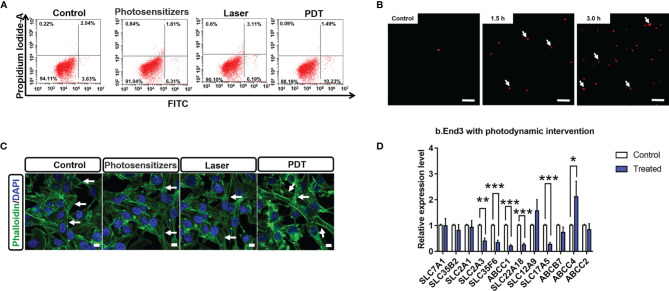
**(A)** Percentage of apoptotic cells stained by Annexin V-FITC and propidium iodide after PDT intervention at 24 h **(B)** Comet assays of B.END3 cells with PDT intervention at 1.5 h and 3.0 h compared to the control group. Nucleus is stained by ethidium bromide. The scale bar is 100 μm. **(C)** Immunostaining for cellular skeletons on control, photosensitizer-treated group, only laser-treated group, and PDT-treated group. Nucleus is stained by DAPI. The scale bar is 50 μm. **(D)** Quantification for mRNA levels of BBB critical biomarkers in B.End3 cells after photodynamic intervention (dose 20 J/cm^2^) (n = 3/group). *p<0.05; **p<0.01; ***p<0.001.

The therapeutic products of PDT are reactive oxygen species (ROS) (29) that can strongly interact with cellular components and affect down-stream gene expression. However, the biological impacts of PDT through ROS are complex. Herein, we firstly examined the impact of PDT-generated ROS on cellular apoptosis, which is a major reason to cause tumor cell death. As shown in **Figure 1A**, we observed that the apoptotic percentage of B.END3 cells after receiving PDT is only 1.49%, similar with control (2.04%) or single-factor interventional group (1.81% and 3.11%, respectively). This result implied that low dosage PDT could not induce cellular apoptosis and corroborate safety on BBB endothelial cells.

Next, we investigated the effects of PDT on DNA and cellular skeleton. To explore the effect of PDT, we performed the comet assay to examine whether PDT intervention caused DNA damage. As shown in **Figure 1B**, fluorescence tails of DNA after PDT did not display any significant change at 1.5 h and 3.0 h in B.END3 cells, because the length of fluorescence tail is the critical index for evaluating DNA damage. These results implied that PDT at 20 J/cm^2^ laser dose and 300 μg/mL porfimer sodium could not directly damage the DNA in cerebrovascular endothelial cells.

The cellular skeleton plays an essential role in supporting cellular structure and further affecting the cellular processes (30). Herein, we investigated whether PDT can affect the structure of B.END3 cellular skeletons by immunostaining method (31) as shown in **Figure 1C**. After intervention, we cannot observe any morphological difference between intervention-treated group and control groups (including sham and single-factor intervention), although the fluorescent intensity of cellular boundary was stronger than that of other groups. These results could be attributed to the cellular stimulus for ROS and may affect the cell migration, requiring further investigation.

The biological integrity of BBB is decided by the compact cell stack and the specific expression of surface biomarkers, i.e. molecular transporters (4). These surface transporters can selectively pump the necessary nutrients into brain parenchyma and the harmful components out of the nervous system (32). To evaluate the impact of PDT on BBB cellular transporters, we quantitatively measured the mRNA expression levels of critical transporters compared to the no intervention group. As shown in **Figure 1D**, we observed that *SLC2A3* (GLUT-3), *SLC35F6*, *ABCC1* (MRP1), *SLC22A18* (efflux transporter-like protein), and *SLC17A5* (acidic Sugar Transporter) are significantly inhibited, while *ABCC4* (MRP4) is significantly up-regulated. The down-regulation of *SLC2A3* and *SLC17A5* implied that PDT might affect the cellular uptake of glucose into brain parenchyma tissue, owing to that these genes are highly associated with glucose uptake (33, 34). *ABCC1*, *ABCC4*, and *SLC22A18* participate in the drug resistance pathway (35). The down-regulation of *ABCC1* and *SLC22A18* implied that PDT improves the BBB uptake of chemotherapeutic drugs, while significant up-regulation of *ABCC4* implied that PDT might blockade the pumping of therapeutic drugs out of nervous system. The comprehensive impacts of PDT on BBB functions and therapeutic drugs are unclear and should be well determined in the future.

### Identification of DEGs

**Figure 2:**
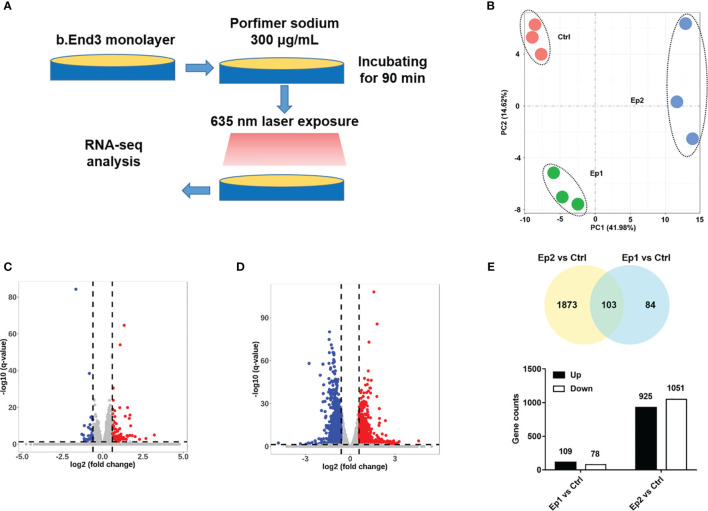
**(A)** Schematic illustration of RNA-sequencing protocol in this investigation. **(B)** PCA analysis of RNA-seq samples based on the genetic components. **(C, D)** Volcano plot of expressed genes based on the intervention dosage. **(E)** Statistical results of different genes (p-adj <0.05 and |log_2_FoldChange|>0.6).

In order to explore the molecular mechanisms of PDT intervention on the cerebrovascular endothelial cells, we performed an RNA sequencing assay to identify the DEGs in the B.END3 cell line. The schematic illustration of the experimental protocol is illustrated in **Figures 2A, B**. END3 cells are seeded dish to form the monolayer and then incubated with photosensitizer (300 μg/mL) for 90 min. Then, the cells were exposed to a 635 nm laser with light doses of 10 J/cm^2^ and 20 J/cm^2^, respectively. RNA-seq assays were employed to identify the genetic profiles after RNA extraction from these cell samples, and the differentially expressed genes were obtained by the comparative transcriptome analysis between PDT-intervention samples and control samples.

Firstly, principal component analysis (PCA) was conducted to analyze the quality of RNA sequencing. As shown in **Figure 2B**, the PCA score of all samples based on the PKFM values of the gene can be divided into three independent subgroups: Ctrl, Ep1, and Ep2, and confirmed that these RNA sequencing data can be employed in the following assays. The relative expression levels of these genes were plotted as a Volcano map (**Figures 2C, D**), and DEGs were highlighted with blue and red colors, respectively. The threshold value considered as the significant difference was set as p-adj <0.05 and |log_2_(foldchange)|>0.6. Herein, we identified 187 and 2976 DEGs in Ep1 and Ep2 groups compared to the control group, respectively. The Venn overlapping diagrams of DEGs in both groups are shown in **Figure 2E**, and 103 joint genes were observed in both groups. Moreover, the significant increase in the DEGs from 187 to 2976 implied that the higher dose of PDT might activate several pathways or biological annotations for PDT stimulus response.

### PPI of DEGs

**Figure 3:**
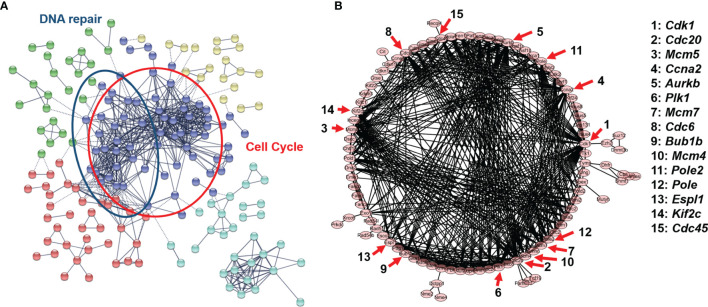
**(A)** Protein-protein interaction network of B.END3 cells with PDT intervention (p-adj<0.05 and |log_2_FoldChange|>1.0). **(B)** Sub-network of cell cycle-related genes and 15-top genes.

To elucidate the stimulus response and identify the hub genes of endothelial cells after PDT treatment, PPI analysis of DEGs was employed (threshold value of significant difference: p-adj <0.05 and |log_2_(foldchange|>1.0). Herein, all the DEGs were uploaded to the STRING website, and 267 interaction nodes were obtained for further analysis. As shown in **Figure 3A**, we found that three major gene clusters were identified, which can be attributed to DNA repair and cell cycle based on GO analysis. In the largest gene cluster, top-ranked 15 genes (**Figure 3B**) were identified based on the degree nodes and relative biological functions were presented in **Table 1**. A total of 14 enriched genes could be attributed to the cellular mitosis process (*CDK1*, *CDC20*, *MCM5*, *MCM7*, *MCM4*, *CCNA2*, *AURKB*, *KIF2C*, *ESPL1*, *BUB1B*) and DNA replication (*POLE2*, *PLOE*, *CDC45*, *CDC6*).

**Table 1:** Biological functions of hub genes in cell cycle-related gene network.

Gene	Biological Function	Gene	Biological Function
CDK1 (DOWN)	Essential for G1/S and G2/M phase transitions of eukaryotic cell cycle	BUB1B (DOWN)	Play a role in the inhibition of the anaphase-promoting complex/cyclosome (APC/C), delaying the onset of anaphase and ensuring proper chromosome segregation
CDC20 (DOWN)	Required for two microtubule-dependent processes, nuclear movement prior to anaphase and chromosome separation	MCM4 (UP)	Essential for the initiation of eukaryotic genome replication
MCM5 (DOWN)	Upregulated in the transition from G0 to G1/S phase of the cell cycle and may actively participate in cell cycle regulation	POLE2 (UP)	Involved in DNA repair and replication
CCNA2 (DOWN)	This protein binds and activates cyclin-dependent kinase 2 and promotes transition through G1/S and G2/M	POLE (DWON)	Involved in DNA repair and chromosomal DNA replication
AURKB (DOWN)	These kinases participate in the regulation of alignment and segregation of chromosomes during mitosis and meiosis by association with microtubules	ESPL1 (DWON)	Stable cohesion between sister chromatids before anaphase and their timely separation during anaphase are critical for chromosome inheritance
PLK1 (DOWN)	Ser/Thr protein kinase and depletion of this protein in cancer cells dramatically inhibited cell proliferation and induced apoptosis	KIF2C (DOWN)	Functions as a microtubule-dependent molecular motor
MCM7 (DOWN)	Essential for the initiation of eukaryotic genome replication	CDC45 (UP)	Essential protein required for the initiation of DNA replication
CDC6 (DOWN)	Essential for the initiation of DNA replication		

DOWN, labeled gene is down-regulated; UP, labeled gene is up-regulated.

For cellular mitosis process-related hub genes, these genes can be attributed to E2F targeted genes and cell cycle pathways. To our knowledge, PDT can up-regulated intracellular ROS levels and induced oxidative stress to further regulate down-stream pathways (36). Higher levels of ROS can activate canonical MAPK pathway, and further regulate E2F-mediated gene transcription by p38/COX2/TGFβ/Rb pathway (37). Enrichment results implied that PDT process may participate into regulation of E2F-mediated gene transcription and further regulate expression of down-stream genes. For these genes, *CDK1* is significantly up-regulated after the H_2_O_2_-induced oxidative stress by inactivating the PI3K/AKT signaling (38); *CCNA2, CDC45*, and *MCM4* are downstream genes of cyclin-dependent kinase inhibitor p16 in D-galactose-induced aging in mice (39); *BUB1B* is involved in cell division and induces the vulnerability for oxidative stress (40); *PLK1*, as the serine/threonine-protein kinase gene, also plays a major role in chromosomal instability (41) and cell cycle progression (42). These hub genes indicated that low-dose PDT intervention induces may participate into regulation of cell cycle-related pathways.

### Impact of Photodynamic Intervention on Transcription Factors

**Figure 4:**
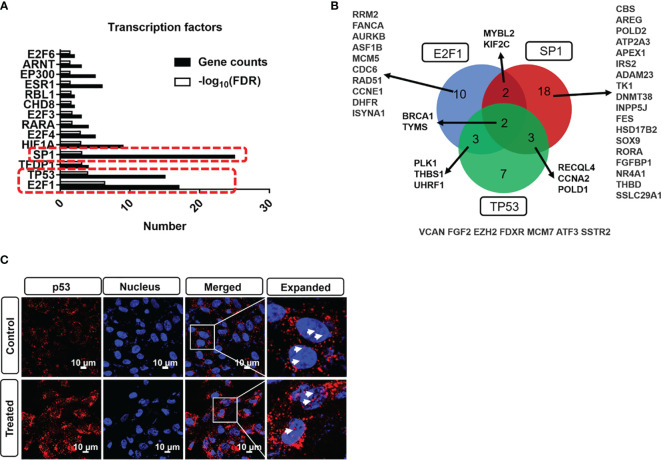
**(A)** Transcription factor profile of DEGs with photodynamic intervention (dose 20 J/cm^2^) using TRRUST database. **(B)** DEGs overlap among E2F1, SP1, and TP53 transcription factors. **(C)** Immunostaining of p53 nuclear translation after photodynamic intervention (20 J/cm^2^). Scale bar is 10 μm.

Before further functional analysis, we explored the transcription factors of the DEGs using TRRUST database. As shown in **Figure 4A**, the top 3 transcription factors of these DEGs are E2F1, TP53, and SP1. By overlapping gene sets based on transcription factors, we can find that several genes are identified as shown in **Figure 4B**. TP53 is one of the critical transcription factors to regulate the expression of ATP binding cassette (ABC) transporter-related genes and further modulate cerebrovascular functions (43). To corroborate the effect of PDT on p53 activity, an immunostaining assay was performed in B.END3 cell line after PDT. As shown in **Figure 4C**, fluorescent staining shows that ROS can significantly promote the nuclear translocation of p53 protein to the nucleus, implying that TP53 plays an essential role in affecting the gene expression of DEGs.

### Functional Analysis of DEGs

To explore the effect of PDT on biological processes, gene ontology (GO) analysis of DEGs was employed. GO analysis of DEGs can provide the scope of molecular mechanism affected by external stimulus, especially for identifying specific pathway to explain how to affect molecular network. Among these GO analysis tools, GO:profiler is a robust tool for functional enrichment analysis using DEGs (44). Herein, we employed go:profiler for GO analysis and obtained the most affected annotations of DEGs in various groups.

For DEGs in Ep1 group compared to control group, the top 15 enriched annotations are listed in **Figure S1**, wherein the cutoff of FDR q-value was set as 0.05. Among these annotations, cellular response-related, endogenous stimulus-related, and vasculature development-related annotations (labeled by red square) are highlighted. These annotations could be attributed to the chemical stimulus, which might originate from ROS stimulus by photodynamic photosensitizer. Among these annotations, oxidative stress might be the major pathway for PDT response due to higher levels of intracellular ROS. To the best of our knowledge, oxidative stress is highly related to vascular diseases (45); for example, participating in nitric oxide pathway in atherosclerosis pathogenesis (46) and inducing inflammation in aging (47). These enriched annotations confirmed the potential damage of PDT intervention on the vessels.

**Figure 5:**
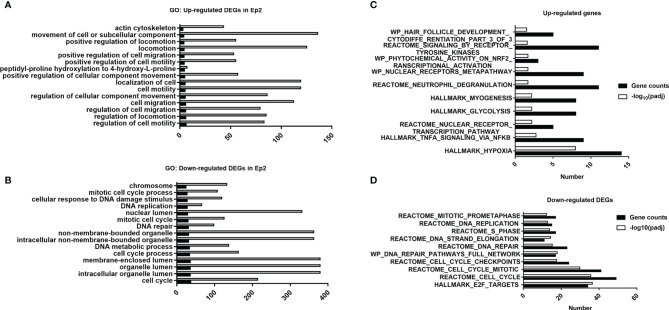
Functional analysis of DEGs in various groups. **(A)** Top 15 annotations of DEGs in the Ep1 group. **(B)** Top 15 annotations of up-regulated DEGs in the Ep2 group. **(C)** Top 15 annotations of down-regulated DEGs in the Ep2 group. **(C)** Computed overlaps between the up-regulated DEGs and MSigDB gene sets. **(D)** Computed overlaps between down-regulated DEGs and MSigDB gene sets. The threshold value considered as the significant difference is p < 0.05 and FDR q-value < 0.05.

When the laser dosage of PDT intervention was increased to 20 J/cm^2^, number of differentially expressed genes were increased to 2976, and these genes could be divided into up- and down-regulated groups (**Figures 5A, B**), which would be utilized for further GO analysis. For up-regulated DEGs, the 15 top enriched GO annotations were annotated in **Figure 5A**, and many annotations about cellular biological functions were enriched. For example, actin cytoskeleton and cell migration-related annotations (i.e., positive regulation of cell migration, cell motility, and location). The migration or motility-related annotations are highly associated with cell skeleton that is regulated by assembly and disassembly of actin filaments (48). These enriched annotations are consistent with the immunostaining data (**Figure 1C**). Moreover, high migration of endothelial cells promotes angiogenesis in tumor tissue (49, 50) and participates in the vascularization process (51). These enriched annotations of up-regulated DEGs implied that PDT intervention might affect the biological functions of endothelial cell skeleton that need to be determined in the future.

For down-regulated DEGs, cell cycle-related annotations (for instance, mitotic cell cycle process, DNA replication, and DNA repair) can be obtained as shown in **Figure 5B**. The down-regulation of these biological pathways indicated that the cell cycle of endothelial cells may be suppressed and cause damage to cellular mitosis process. Moreover, the enrichment of chromosome, DNA repair, and DNA metabolic process confirmed that the PDT process damaged the DNA. The suppression of organelle-related annotations (non-membrane-bounded organelle, lumen-related annotations) indicated that PDT might damage nucleoplasm. However, we did not observe any significant impact of PDT on cell apoptosis (**Figure 1A**). All the enriched GO terms are related to the translation process and cell cycle. As reported previously, photodynamic therapy induces cellular autophagy to prevent cellular apoptosis (23). These findings implied that PDT might activate cellular autophagy against the exogenous stimulus.

### GSEA Analysis

GO and PPI analysis of DEGs could predict the potential impact on the cellular biological processes. However, it is difficult to identify the attribution of PDT to specific pathways. Conversely, GSEA, which ranked all genes based on the expression level, can be employed to evaluate roles of DEGs on targeted pathways (52). Herein, we employed GSEA to identify critical pathways affected by PDT, including Hallmark, KEGG, Wikipathways, and PID pathway gene sets.

Before the GSEA scoring analysis, we firstly analyzed the overlaps between DEGs and pathway gene sets, which can be divided into up- and down-regulated DEGs, and top 10 ranked pathways are listed in **Figures 5C, D**. For up-regulated DEGs, the top 10 ranked pathways were Hallmark_hypoxia, Hallmark_TNFA_signaling_*via*_NFKB, Reactome_nuclear_receptor_trascritpion_pathway, Hallmark_glycolysis, Hallmark_myogenesis, Reactome_neutrophil_degranulation, WP_nuclear_receptor_metapathway, WP_phyochemical_activity_on_nrf2_transcription_activation, Reactome_signaling_by_receptor_tyrosine_kinases, and WP_hair_follicle_development_cytodiffe_rentiation_part_3_of_3. For down-regulated DEGs, top 10 ranked pathways were Hall_E2F_targets, Reactme_cell_cycle, Reactome_cll_cycle_mitotic, Reactome_cell_cycle_checkpoints, WP_DNA_repair_pathways_full_network, Reactome_DNA_repair, Reactome_DNA_strand_elongation, Reactome_S_phase, Reactome_DNA_replication and Reactome_mitotic_prometaphase. These results suggested that major pathways affected by PDT may focus on inflammation response and cell cycle regulation, which is consistent with GO analysis.

**Figure 6:**
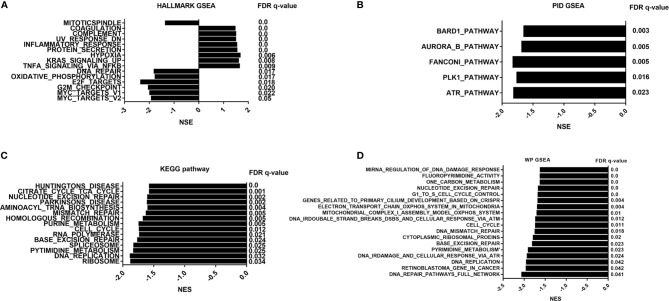
GSEA pathway analysis of B.END3 cells treated with PDT intervention. **(A)** Hallmark gene set analysis; **(B)** PID pathway gene set analysis; **(C)** KEGG pathway gene set analysis; **(D)** WikiPathways gene set analysis. The threshold value considered as the significant difference is P < 0.05, FDR q-value < 0.05, and |NES|>1.0.

To address the status of critical pathways after PDT treatment, GSEA plots were performed using Hallmark, KEGG, Wikipathways, and PID pathway gene sets (Normalized enriched score, |NES|>1.0 and NOM p-value < 0.05 and FDR q-value <0.05). However, we did not obtain any GSEA terms in Ep1 group compared to control group. Subsequently, only GSEA results of Ep2 group compared to control group were analyzed. As shown in **Figure 6**, only 8 pathways are significantly up-regulated (NES>1.0): coagulation, complement, UV response, inflammatory response, protein secretion, hypoxia, KRAS, and TNFA signaling *via* NFKB. The activation of *KRAS signaling* in endothelial cells induces ERK activity and promotes the expression of angiogenesis and notch signaling, which enhances the cell migration (53). *Coagulation* term means regulation of the blood coagulation system, which is also related to platelets (54). *Hypoxia* is always up-regulated and under low oxygen conditions. *Complement*, *inflammatory response*, and *TNFA signaling via NF-κB* is the major immunological response for the exogenous stimulus that is related to the vasculature disease (55). As above-mentioned assays, expression levels of several transporter-related genes were significantly down-regulated, except *ABCC4*. As previous reported, inflammation response can suppress the expression of ABC-related transporters through affecting Toll-like receptors (56). Moreover, inflammation-related stimulus can promote the expression of MRP4 (*ABCC4*) through ROS/NF-κB pathway (57). Moreover, NES value of 48 GSEA terms was less than -1.0, which implied the down-regulation of these pathways, and these annotations were highly associated with cell cycle process. As previous reported, ROS can regulate cell cycle by p38/ERK MAPK (58) or Cdc25C activity (59). As a result, these results indicated that PDT intervention can affect BBB function through inflammatory response, i.e. *TNFA signaling via NF-κB*.

**Figure 7:**
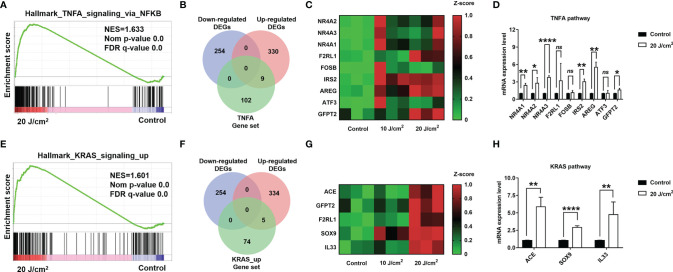
**(A)** Enrichment score profile of enriched pathway, TNFA_signaling_*via*_NFKB. **(B)** Venn diagram of overlapped genes among DEGs and leading edge gene set. **(C)** Heatmap of overlapped gene expression level. **(D)** Quantification for mRNA levels of DEGs in TNF-α/NF-κB pathway (dose 20 J/cm^2^). **(E)** Enrichment score profile of enriched pathway, KRAS_signaling_up. **(F)** Venn diagram of overlapped genes among DEGs and leading-edge gene set. **(G)** Heatmap of overlapped gene expression level. **(H)** Quantification for mRNA levels of DEGs in KRAS_signaling_up pathway (dose 20 J/cm^2^). *p<0.05; **p<0.01; ****p<0.0001; ns, no significance.

To further determine whether PDT treatment can up-regulate *TNFA signaling via NF-κB* and *KRAS* pathways, we utilized qRT-PCR to examine expression levels of critical genes in these pathways. Firstly, we identified the critical genes by overlapping DEGs and pathway gene sets, i.e. *TNF-α signaling via NF-κB* and *KRAS* signaling pathways (**Figures 7A–H**), and these critical genes included *NR4A1, NR4A2, NR4A3, F2RL1, FOSB, IRS2, AREG, ATF3, GFPT2*, *ACE*, *SOX9*, and *IL33*. The qRT-PCR assays confirmed the up-regulation of *NR4A1, NR4A2, NR4A3, IRS2, AREG, GFPT2, ACE, SOX9*, and *IL33*, respectively. These results demonstrated that *TNF-α/NF-κB* and *KRAS* pathways were substantially up-regulated after PDT intervention.

## Conclusion

In summary, we built one approach of RNA sequencing to well-understand the effect of photodynamic intervention in cerebrovascular endothelial cells at cellular transcriptome level. These results provide essential information to elucidate the stimulus response of endothelial cells receiving PDT intervention, which might be associated with affecting the expression of BBB endothelial transporters by activating inflammatory response pathways and cell cycle-related pathways. This stimulus response is crucial for the normal cerebrovascular endothelial cells and to maintain BBB homeostasis. Thus, we speculated that the current study could guide the clinical application of PDT in nervous system diseases and further decrease the drawback of PDT intervention on nervous functions.

## Data Availability Statement

The datasets presented in this study can be found in online repositories. The names of the repository/repositories and accession number(s) can be found below: https://www.ncbi.nlm.nih.gov/, GSE172198.

## Author Contributions

YKH and TXL: Conceptualization, Methodology, Funding acquisition, Supervision. YYH: Investigation, Conceptualization, Methodology, Funding acquisition, Writing – original draft preparation, Resources. LD and HW: Investigation, Writing – original draft preparation, Writing – review & editing. SC and TYL: Methodology, Writing – review & editing, Resources.

## Funding

This study was supported by funding from Henan Province Excellent Young Talents Training Project (YXKC2020041) and the Key Scientific And Technological Project of Henan Province (202102310037).

## Conflict of Interest

The authors declare that the research was conducted in the absence of any commercial or financial relationships that could be construed as a potential conflict of interest.

## Publisher’s Note

All claims expressed in this article are solely those of the authors and do not necessarily represent those of their affiliated organizations, or those of the publisher, the editors and the reviewers. Any product that may be evaluated in this article, or claim that may be made by its manufacturer, is not guaranteed or endorsed by the publisher.
