# Supplementary material for: Integrated Transcriptome Analysis Reveals the Impact of Photodynamic Therapy on Cerebrovascular Endothelial Cells
Source: Front Oncol. 2021 Nov 22;11:731414. doi: 10.3389/fonc.2021.731414 (PMC8645902; doi:10.3389/fonc.2021.731414)
Supplement: Supplementary file 1 [file DataSheet_1.docx]

SI Table 1. Primer sequence used in this study

|  | Forward primer | Reverse primer |
| --- | --- | --- |
| ACE | TCCCAGTCCAGACAGTCTCC | CCAGGAAATTGACGCGGTTG |
| NR4A1 | CTGCGAAAGTTGGGGGAGT | CTTGAATACAGGGCATCTCCAG |
| NR4A2 | GAGCTGGAGCTGGGCTG | ACAAGGCATGGCTTCAGCA |
| NR4A3 | GGATGCCTGCGGAACCTC | CGCAGTGGGCTTTGGGTG |
| F2RL1 | GAGTAGGGCTCCGAGTTTCG | CTGTTGTTGCGTCCCGGTG |
| FOSB | CAGCTAAGTGCAGGAACCGT | ACTTGAACTTCACTCGGCCA |
| IRS2 | GAGACAAGAACTCCTGGAGCC | TAGCGCTTCACTCTTTCACG |
| AREG | GCTGAGGACAATGCAGGGTAA | AGTGACAACTGGGCATCTGG |
| ATF3 | AGCGAAGACTGGAGCAAAATG | CATCCGATGGCAGAGGTGTT |
| GFPT2 | CCACCATGTGCGGAATCTTT | CAACCCCCGCAGAGTCATAG |
| SOX9 | CACAAGAAAGACCACCCCGA | GGACCCTGAGATTGCCCAGA |
| IL33 | GGCAGAATCATCGAGAAAAGGC | TTTGCCGGGGAAATCTTGGA |
|  |  |  |
| SLC7A1 | GCTCTTTCCGCCAGTCTTCT | AAGAGGAAGGCCATCACAGC |
| SLC35B2 | CACAGGGCTCCAGGTGTC | TTAGCACCAGGAACTGCGAG |
| SLC2A1 | CACTGTCGTGTCGCTGTTTG | AAAGATGGCCACGATGCTCA |
| SLC2A3 | ATCCTTCCTGAGGACGTGGAG | TATCAGAGCTGGGGTGACCTTC |
| SLC35F6 | CACGCTCTCGGCAAAATGG | TACGCTGGAGTCTGATTGCC |
| ABCC1 | GGTGGACGAGAACCAGAAGG | TCAAGTACGTGGTGACCTGC |
| SLC22A18 | CTGCAAAGGCAGGCAAGG | GAGAGATCCAAGCAGGAGGC |
| SLC12A9 | GTAGCCGCAGCTGTCTTTTC | AAATGGGTTAGGTGACCCGC |
| SLC17A5 | AGCATTTCATATGCAGGAGCAC | TGTGTTTTTGTGGTGTGTCACT |
| ABCB7 | TCGAGCCTACCAGCAGATTC | TCAGTGGCCATACCTGGAGA |
| MRP4(ABCC4) | CGTGTTCTTCTGGTGGCTCA | CTCCTCTCCAAGGTGCTGTG |
| BCRP1(ABCC2) | GACTTATGTTCCACGGGCCT | GGCTCTATGATCTCTGTGGCTTT |
| ACTIN | CTACAATGAGCTGCGTGTGG | AAGGAAGGCTGGAAGAGTGC |


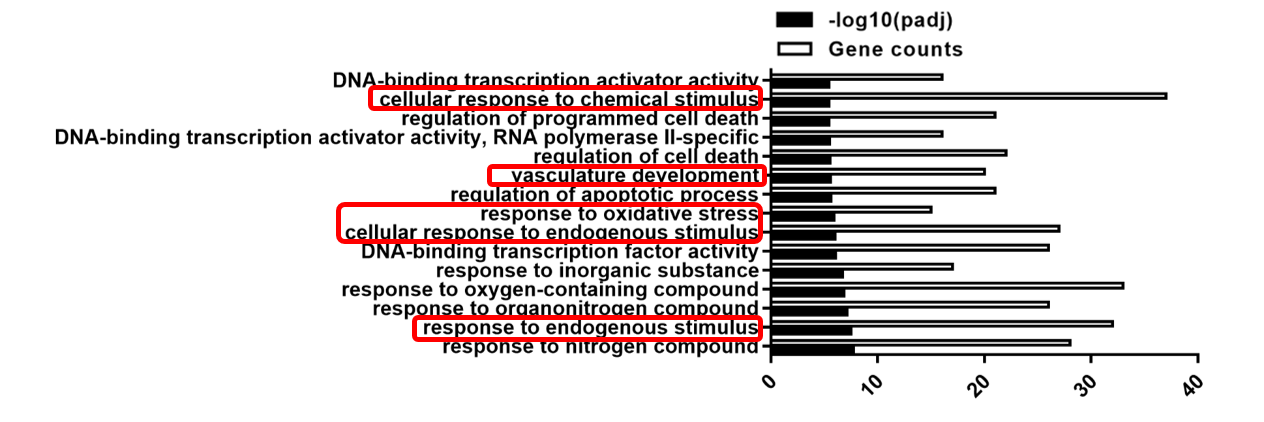


SI Figure S1. GO analysis of DEGs in Ep1 vs control groups. Here, we listed the top 15 annotations.
